# *Bifidobacterium* BLa80 mitigates colitis by altering gut microbiota and alleviating inflammation

**DOI:** 10.1186/s13568-022-01411-z

**Published:** 2022-06-07

**Authors:** Yao Dong, Wenyan Liao, Jing Tang, Teng Fei, Zhonghui Gai, Mei Han

**Affiliations:** 1 Shanghai Business School, 2271# Zhongshanxilu Road, Shanghai, 200235 China; 2 State Key Laboratory of Dairy Biotechnology, Technology Center Bright Dairy & Food Co., Ltd., Shanghai, 200436 China; 3 Department of Research and Development, Wecare-Bio Probiotics (Suzhou) Co., Ltd., Wujiang Bridge Road, 1033, Suzhou, Jiangsu China

**Keywords:** Inflammatory bowel disease, Ulcerative colitis, Probiotic, Proinflammatory cytokines, Gut microbiota

## Abstract

This study was conducted to explore the therapeutic effect of the probiotic *Bifidobacterium animalis* subsp. *lactis* BLa80 on inflammatory bowel disease. A model of ulcerative colitis (UC) was induced in C57BL/6 mice by administering of 2.5% dextran sulphate sodium (DSS) for 8 days. After developing UC, some mice were treated via intragastric administration of BLa80 at a dose of 10^9^ colony-forming units to assess the preventive effects of BLa80 on DSS-induced UC. Compared with non-treated UC model mice, BLa80-treated mice had reduced colon shortening and improvements in colonic tissue structure. Treatment with BLa80 also decreased the serum concentrations of the proinflammatory cytokines tumor necrosis factor-alpha (TNF-α), interleukin (IL) 6 and IL-17 in mice. 16S rRNA gene sequencing revealed that BLa80 increased gut microbial diversity in mice and modulated UC-associated imbalances in the gut microbiota. BLa80 selectively promoted the growth of beneficial bacteria, including *Romboutsia* and *Adlercreutzia*, the abundances of which were negatively correlated with concentration of cellular inflammatory factors. In summary, the study results demonstrated that pretreatment with *B*. *lactis* BLa80 reduced intestinal inflammation and altered the gut microbiota, implying that BLa80 is a promising probiotic strain with potential therapeutic function in UC.

## Introduction

Inflammatory bowel disease (IBD) is a group of chronic diseases characterized by gastrointestinal inflammation, including Crohn’s disease and ulcerative colitis (UC) (Debnath et al. 2013; Norouzinia et al. 2017). Classically, UC affects the lining of the rectum or large intestine and may cause frequent diarrhoea, abdominal cramping, and rectal bleeding (Debnath et al. 2013). Although the aetiology of IBD remains uncertain, disruption of the intestinal mucosal immune system, defects in the intestinal mucosal barrier, and some genetic and environmental factors have been implicated (Khor et al. 2011). Immunosuppressive drugs, such as 5-aminosalicylic acid, corticosteroids, methotrexate, and thiopurines, are the main medications used in the treatment of IBD (Abreu 2002; Cheifetz 2013). However, these drugs usually provide inadequate treatment and occasionally cause serious adverse effects (Lakatos and Lakatos 2008; Sergent et al. 2010). Therefore, novel alternatives for IBD prevention and clinical treatment are needed (Sergent et al. 2010).

Probiotics are defined as ‘live microorganisms that, when administered in adequate amounts, provide a health benefit on the host’ (Hill et al. 2014). Some probiotic strains belonging to the genera *Bifidobacterium* and *Lactobacillus* have been reported to reduce the symptoms of IBD (Chae et al. 2018; Kumar et al. 2017; Logan and Katzman 2005; Shadnoush et al. 2013; Xie et al. 2017). Probiotics play beneficial roles in the host, e.g., they inhibit the growth of pathogenic microorganisms in gut microflora, promote the production of substances that contribute to cell proliferation and maturation, correct imbalances in the intestinal microflora, and regulate immunity (Gonçalves et al. 2018; Howarth 2008; Neu 2019). Given the correlation between IBD and the gut microbiota, prevention strategies that target an abnormal gut microbiota composition may be effective. Studies have shown that probiotic species of genera such as *Bifidobacterium* and *Lactobacillus* can effectively reduce populations of pathogenic gut microbes in patients with IBD, thereby alleviating dysbacteriosis (Aggeletopoulou et al. 2019; Khan et al. 2019). However, the beneficial effects of probiotics are species- or even strain-specific, and new probiotics are needed to support the use of it in patients with IBD (Veerappan et al. 2012).

Dextran sulphate sodium (DSS) induction is a common chemical method used to induce intestinal inflammation (e.g., colitis) in animals. Epithelial cell death caused by DSS may impair intestinal barrier function and contribute to subsequent inflammation. Animal models of DSS-induced UC enable studies of epithelial barrier function and innate immune responses (Singh et al. 2018). In rodents, DSS-induced colitis results in a major shift in the gut microbiota, which is similar to changes occurring in human patients with UC (Wang et al. 2017).

*Bifidobacterium animalis* subsp. *lactis* BLa80 is a commercial strain produced by Wecare probiotics (Suzhou) Co., Ltd. Recent studies have shown that compound probiotics containing BLa80 improved survival time, reduced intestinal epithelial damage, and partially restored diversity of intestinal microbiota in mice exposed to total body irradiation (dose = 9 Gy) (Zhao et al. 2021). The purpose of this study was to further assess whether *B*. *lactis* BLa80 could attenuate the severity of DSS-induced colitis in mice.

## Materials and methods

### Preparation of *B*. *lactis* BLa80

BLa80 was provided by Wecare-bio Probiotics (Suzhou) Co., Ltd. DeMan-Rogosa-Sharpe (MRS) agar was purchased from Hopebio Co., Ltd (Qingdao, China). BLa80 was cultured using de Man-Rogosa-Sharpe (MRS) broth at 37 °C for 24 h in an anaerobic environment, the fermentation pH was controlled around 5.5 by feeding 20% NaOH. Then, bacterial cells were collected by centrifugation at 7000×*g* for 15 min, and the collected cells were washed twice with sterile saline and resuspended. The bacterial solution was freshly prepared before each administration to mice.

### Animals and treatment

Dextran sulphate sodium (DSS, MW: 36,000–50,000 Da) was obtained from Yeasen Biotechnology (Shanghai) Co., Ltd. Male C57BL/6 J mice (4 weeks old) were purchased from Shanghai Laboratory Animal Research Center (Shanghai, China). Ethical approval for all animal experimental procedures was provided by the Animal Ethics Committee of Shanghai Laboratory Animal Research Center (Ethics No. 2021082003). Mice were kept in a room with a controlled light schedule (12-h light–dark cycle) and temperature (25 ± 2 °C) with free access to food and water throughout the study. After a 7-day adaptive feeding period prior to study initiation, the mice were randomly divided into three groups (n = 10/group): the control (CTL) group, UC group and BLa80 group. Mice in the CTL group were given tap water; while those in the UC and BLa80 groups were given tap water with DSS (2.5%). Mice in the BLa80 intervention group were administered 10^9^ CFU of BLa80 daily intragastrically by gavage from the next day of DSS administration, while mice in the CTL and UC groups were administered an equal volume of normal saline by gavage. The duration of the modelling and intervention process was 8 days, after which mice in all of the three groups were given untreated drinking water for the rest 4 days.

### Evaluation of Disease Activity Index (DAI)

UC was evaluated using the Disease Activity Index (DAI), which is based on based on body weight, stool consistency, and the presence of occult blood in the stool (Marchesi et al. 2007). The DAI was calculated every 3 days from the beginning of DSS induction to the end of the study. The mice were weighed daily, and the following clinical scores were assessed. Weight loss was scored as: 0, no weight loss, 1, 1–5% reduction; 2, 5–10% reduction; 3, 10–15% reduction; or 4,  > 15% reduction. Stool consistency was scored as: 0, well-formed pellets; 2, pasty and semi-formed feces not adhering to the anus; or 4, watery diarrhea adhering to the anus. Intestinal bleeding was scored as 0, blood occult negative, 2, blood occult positive, or 4, major bleeding.

### Histological analysis

At the end of the experiment, tissue samples were collected from the distal colon of each mouse, washed with phosphate buffer saline, cut longitudinally and fixed overnight using 4% paraformaldehyde. Then, the distal colon tissues were dehydrated using in a graded ethanol series, embedded in paraffin, and stained with hematoxylin and eosin (H&E). The tissues were observed under a light microscope to detect histological damage. The severity of UC was assessed based on the histological scores, which were based on histopathological morphology (Hassan and Hassan 2018).

### Analysis of serum biochemical parameters

At the end of the experiment, the blood samples collected from each mouse were centrifuged at 4000*g* for 10 min at 4 °C to collect serum. The serum concentrations of tumor necrosis factor alpha (TNF-α), Interleukin (IL)-6, and IL-17 factors were measured using enzyme-linked immunosorbent assay (ELISA) kits following the manufacturer’s instructions (Nanjing Jiancheng Bioengineering Institute, Nanjing, China).

### Microbiota analysis

Colonic stool samples were collected from the mice for intestinal microbiota analysis at the end of the experiment. Total DNA was extracted from 200 mg of feces using the QIAamp Fecal DNA Extraction Kit (Qiagen). The 16S rRNA V3-V4 region was amplified by PCR using 341F (5′-CCTACGGGNGGCWGCAG-3′) and 805R (5′-GACTACHVGGGTATCTAATCC-3′) primers. The final 16S rRNA gene amplicon library was sequenced on the MiSeq platform (Illumina) using a 2 × 300 bp paired-end protocol. The obtained paired-end reads were merged using Usearch11 (https://www.drive5.com/usearch/) and reads ≥ 400 pb in length were retained for subsequent analysis. All quality-filtered sequences were mapped to chimera-free amplicon sequence variants (ASVs), and an ASV abundance table was created using USEARCH11 with the default settings. The phylogenetic assignment of representative sequences for each ASV was determined using the Usearch SINTAX algorithm (Edgar 2016), with the 16S rRNA database of the RDP training set (v18 version) as the reference database (https://www.drive5.com/usearch/manual/sintax_downloads.html). Based on the ASV abundance table, the α diversity indices of chao1, Shannon_e, Berger_Parker, and Simpson diversity was calculated with Usearch alpha_div. *beta*-diversity was assessed at ASV level with principal coordinates analysis (PCoA, Bray–Curtis distance algorithm) and permutational multivariate analysis of variance using the adonis function of R package vegan (Oksanen et al. 2019).

Linear discriminant analysis effect size (LEfSe) was used to identify biomarkers characteristic of each group based on ASV table (Segata et al. 2011). To identify differentially abundant functional processes and pathways among the three groups, microbial pathway abundance was predicted based on taxonomic profiles obtained from 16S rRNA gene amplicon sequencing data (Langille et al. 2013), using the picrust2_pipeline.py script in Picrust2 (version 2.5). Statistical analysis and visualisation of the Picrust2 analysis results were performed using the Statistical Analysis of Metagenomes and Other Profiles package, version 2.1.3 (Parks et al. 2014).

### Statistical analysis

Quantitative data are expressed as the arithmetic mean ± standard deviation (SD) for each group. The effect of treatment was determined by one-way analysis of variance (ANOVA) and differences between treatments were analyzed post-hoc using Tukey’s honest significant difference test. P-values < 0.05 were considered to indicate statistical difference. Data visualization was conducted using ggplot2 on the R platform (Wickham 2017). R version 4.1 was used to perform all statistical tests (Team RC 2013).

### Availability of data and materials

The 16S rRNA sequencing data have been deposited in the Sequence Read Archive database of the National Center for Biotechnology Information under the Accession ID Number PRJNA769551.

## Results

### Effect of *B. lactis* BLa80 on DSS-induced colitis symptoms

**Fig. 1:**
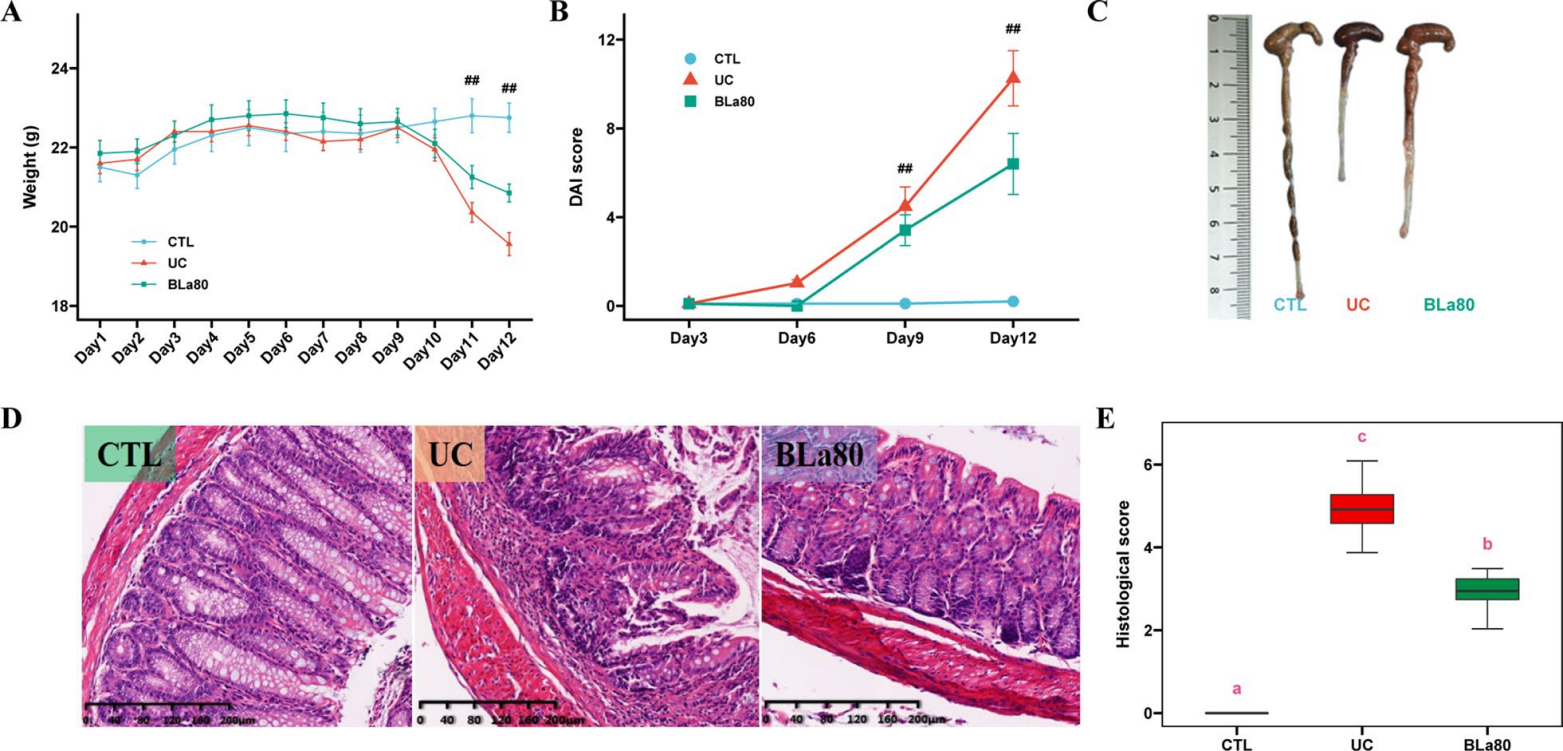
Effect of BLa80 on body weight, DAI, colon length and distal colon tissue in UC mice. BLa80 significantly improved UC physiological parameters in mice: **A** body weight change in mice; **B** DAI assessment of UC in mice; the above data were characterized as mean ± standard deviation (SD). **C** Macroscopic picture of colon length; **D** Pathological examination of colon tissue (scalebar = 200 μm); **E** histological score

The protective effect of *B. lactis* BLa80 in mice was assessed using the body weight, DAI, colon length, and histological examination of the distal colon. As shown in Fig. 1A, DSS administration resulted in a significant decrease in the body weight of mice in the UC group on days 11 and 12. Although mice in the BLa80 group also exhibited body weight losses, the decreases were significantly smaller than those in the UC group. As shown in Fig. 1B, the DAI scores of mice in the UC group increased significantly, whereas those of mice in the CTL group remained at 0 throughout the experiment. Importantly, the intervention with *B. lactis* BLa80 significantly reduced the DAI score compared with UC group. Mice in the UC group exhibited a reduction in colon length, whereas treatment with *B. lactis* BLa80 reversed DSS-induced colon shortening (Fig. 1C). Histological examination of the distal colon showed severe mucosal inflammation in the UC group, which typically was accompanied by crypt damage, infiltration, ulceration, and oedema in the intestinal epithelial layer. These pathological changes in the colon were alleviated in the BLa80 group (Fig. 1D).

### *B. lactis* BLa80 reduces inflammation in UC mice

**Fig. 2:**
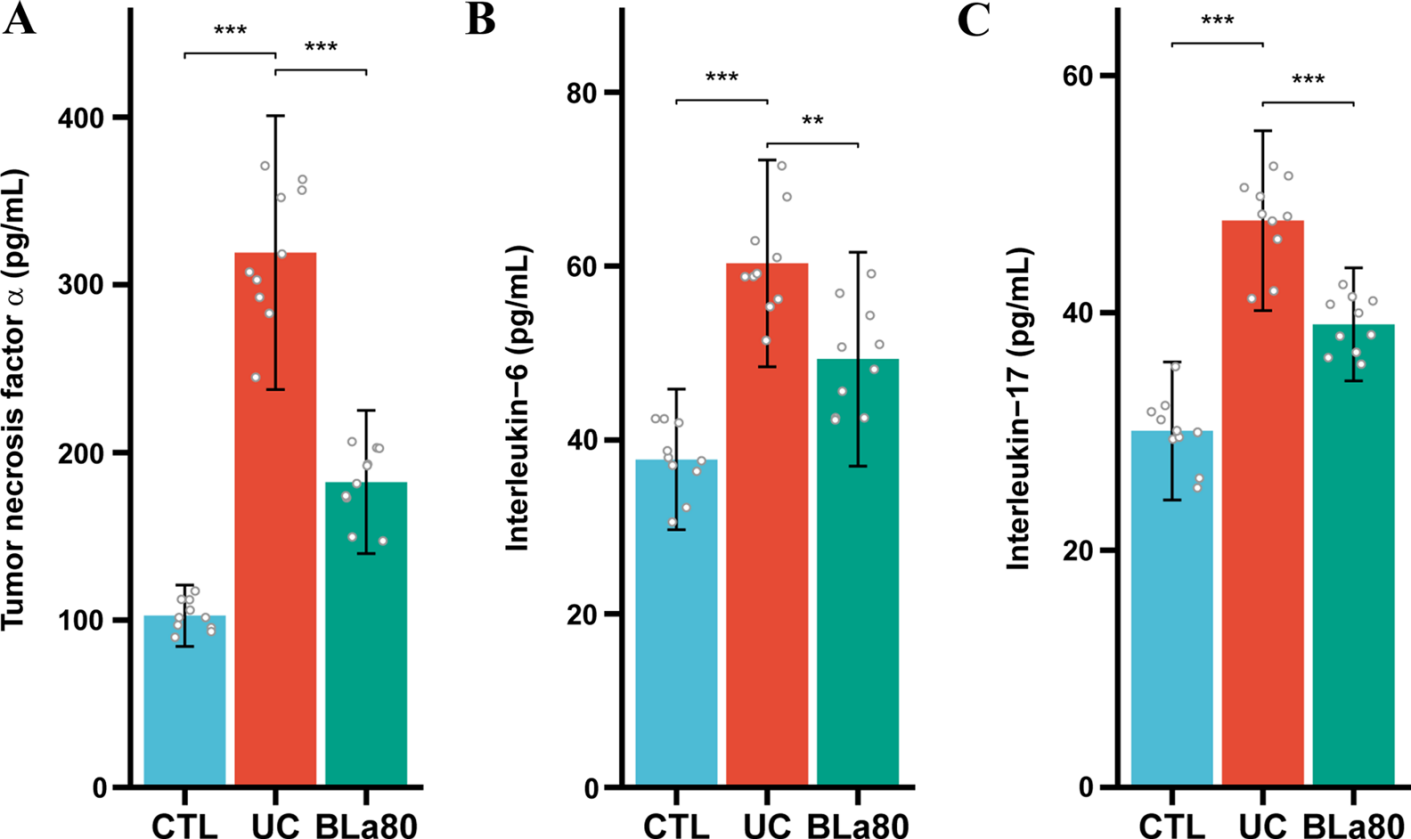
Effect of Bla80 on serum cytokine levels in mice. **A** serum tumor necrosis factor α (TNFα) level in mice; **B** serum Interleukin (IL) 6 level in mice; **C** serum IL17 level in mice. Compared with the model group, BLa80 significantly decreased the serum levels of TNFα, IL6 and IL17

The effect of *B. lactis* BLa80 on systemic indicators of inflammation in the colonic mucosa of mice with DSS-induced UC is demonstrated in Fig. 2. The serum concentrations of the proinflammatory cytokines TNF-α, IL-6, and IL-17 were significantly increased in the UC group compared with the CTL group, but were dramatically reduced in the BLa80 group compared with the UC group.

### Effect of *B. lactis* BLa80 on the gut microbiota in mice with DSS-induced UC

**Fig. 3:**
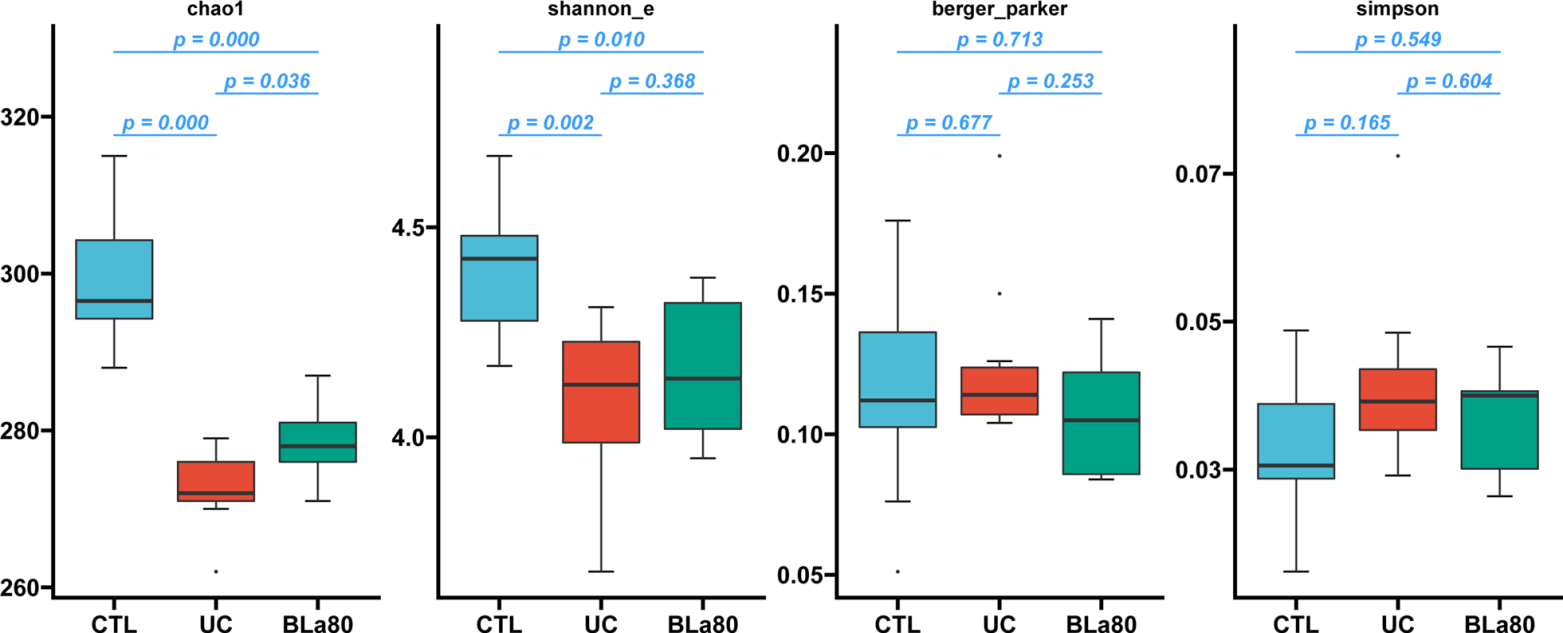
Effect of BLa80 on the α diversity of gut microbiota. No differences were detected in the diversity (shannon_e, berger_perker, and simpson metrics) but richness (Chao1 index) in the GI microbiota increased in the Bla80 group

The gut microbiota of mice in the CTL, UC, and BLa80 groups were detected by high-throughput pyrosequencing of the V3-V4 region of 16S rDNA. As shown in Fig. 3, the α diversity of the microbial community in the UC group decreased significantly in terms of microbial richness (Chao1 index) and microbial diversity (Shannon index). Compared with the UC group, the BLa80 group increased the microbial richness of the microbial community, but there was no significant difference on the microbial diversity between the two groups.

**Fig. 4:**
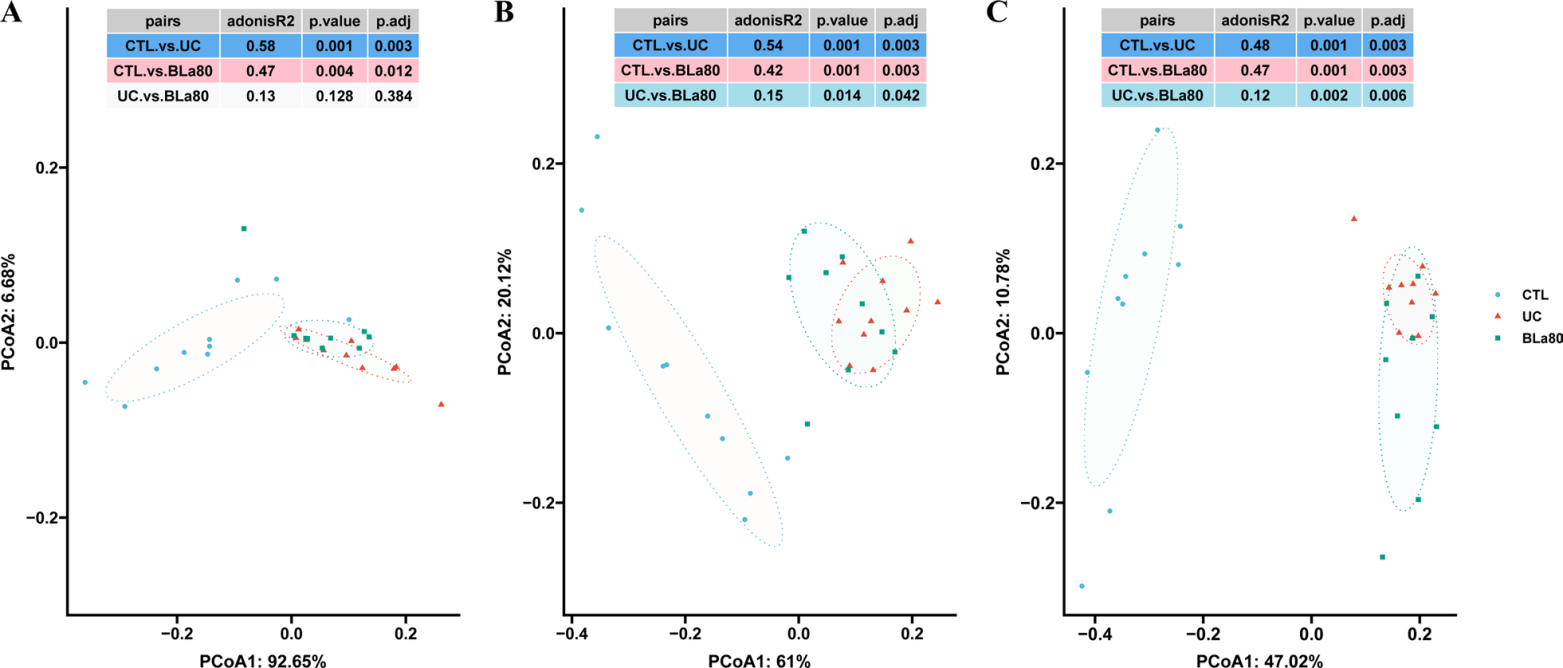
Effect of BLa80 on gut microbiota in UC mice. The composition distribution patterns under different taxonomic classifications including phylum (**A**), family (**B**) and genus (**C**) levels were compared using PCoA. We also performed a significance analysis using pairwise adonis

The distribution patterns of principal components of the gut community structure of mice in each group at different taxonomic levels (phylum, family and genus) were compared via PCoA, and the significance of differences in community structure was tested using pairwise adonis analysis. As shown in Fig. 4, the paired adonis test showed that the samples clearly clustered into three groups at the family and genus levels. This finding illustrates that *B. lactis* BLa80 altered the structure of the gut microbiota in mice with DSS-induced UC.

**Fig. 5:**
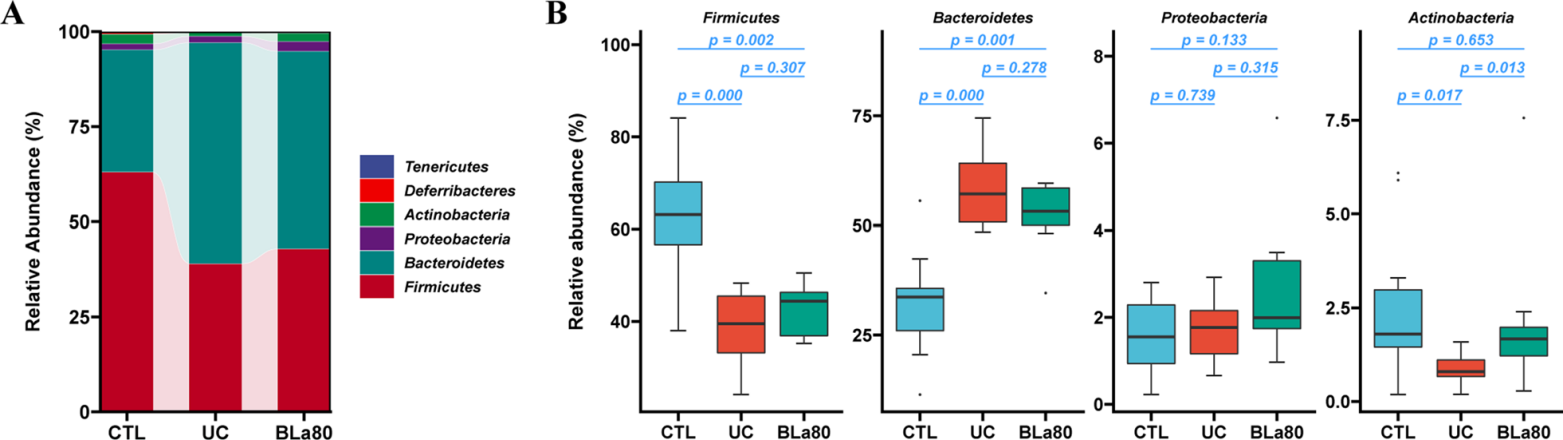
Effect of BLa80 intervention on the abundance of gut microbiota in UC mice. Alluvial plot (**A**) and changes in abundance (**B**) at the phylum level

At the phylum level, composition analysis revealed that *Firmicutes* and *Bacteroidetes* were predominant in the gut microbiota of all three groups of mice, although the relative abundances differed between the groups (Fig. 5A). Compared with the CTL group, the UC group had an increased relative abundance of Bacteroidetes and decreased abundances of *Firmicutes* and *Deferribacteres*. No significant differences in the abundances of *Bacteroidetes* and *Firmicutes* were observed in the BLa80 group, whereas this group had an increased relative abundance of *Actinobacteria* compared with UC group (Fig. 5B).

**Fig. 6:**
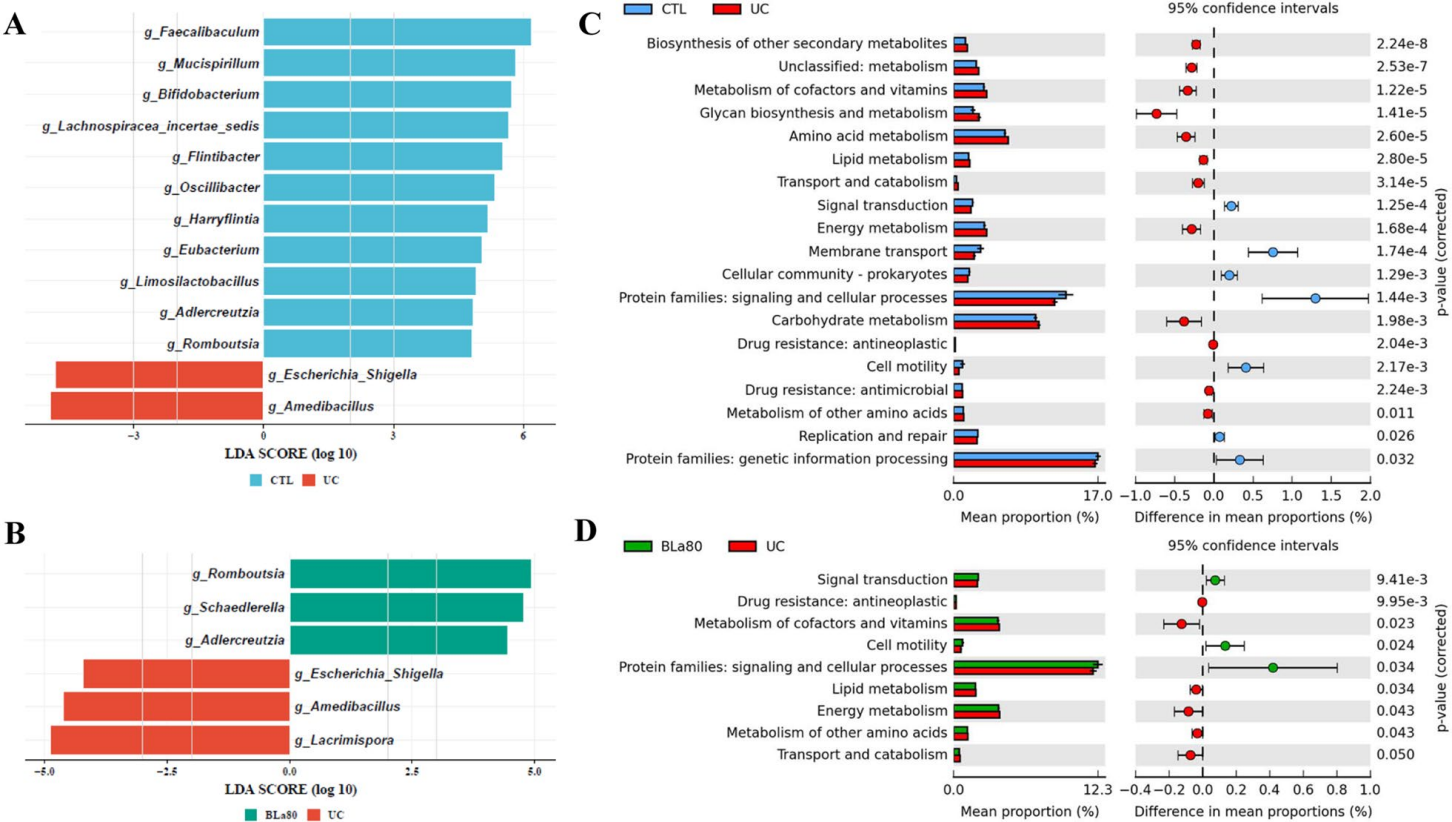
Metagenomic biomarker discovery (LEfSe) and PICRUSt functional analyses. Differential enriched bacterial taxa at the genus level in gut microbiota of mice after DSS treatment (**A** CTL vs UC) and after BLa80 intervention (**B** UC vs BLa80). Extended error bar plots representing microbial pathways predicted to be differentially enriched after DSS treatment (**C** CTL vs UC) and microbial pathways predicted to be differentially enriched after BLa80 intervention (**D** UC vs BLa80). For LEfSe analysis, the threshold LDA score was 2.0

LEfSe analysis showed differential enrichment of genera in the CTL, UC, and BLa80 groups. Compared with the CTL group, mice in the UC group showed increases in the abundances of *Escherichia/Shigella* and *Amedibacillus*, and decreases in the abundances of *Faecalibaculum*, *Bifidobacterium*, *Eubacterium*, *Adlercreutzia*, and *Romboutsia* (Fig. 6A)*.* BLa90 intervention had no significant effect on the abundance of *Escherichia/Shigella*. However, significant increases in the abundances of strains belonging to the genera *Romboutsia* and *Adlercreutzia* were demonstrated in the BLa80 group (Fig. 6B). However, the abundance of *Escherichia/Shigella* did not change significantly after BLa80 intervention. Additionally, a significant increase in the abundance of strains belonging to the genera *Romboutsia* and *Adlercreutzia* was demonstrated in the BLa80 intervention group. (Fig. 6B).

PICRUSt analysis was used to predict how BLa80 intervention might affect functional pathways. Compared with the CTL group, predicted microbial functions such as carbohydrate metabolism, glycan biosynthesis and metabolism, amino acid metabolism, metabolism of cofactors and vitamins, and energy metabolism were predicted to increase in mice in the UC group, whereas protein families: signaling and cellular processes, membrane transport, protein families: genetic information processing, and cell metabolism were predicted to decrease (Fig. 6C). After BLa80 intervention, this trend was partially reversed, as shown by the predicted significant increases in microbial functions such as protein families: signaling and cellular processes and cell motility and decreases in the metabolism of cofactors and vitamins, lipid metabolism, energy metabolism, metabolism of other amino acids and transport and catabolism (Fig. 6D).

### Correlation analysis

**Fig. 7:**
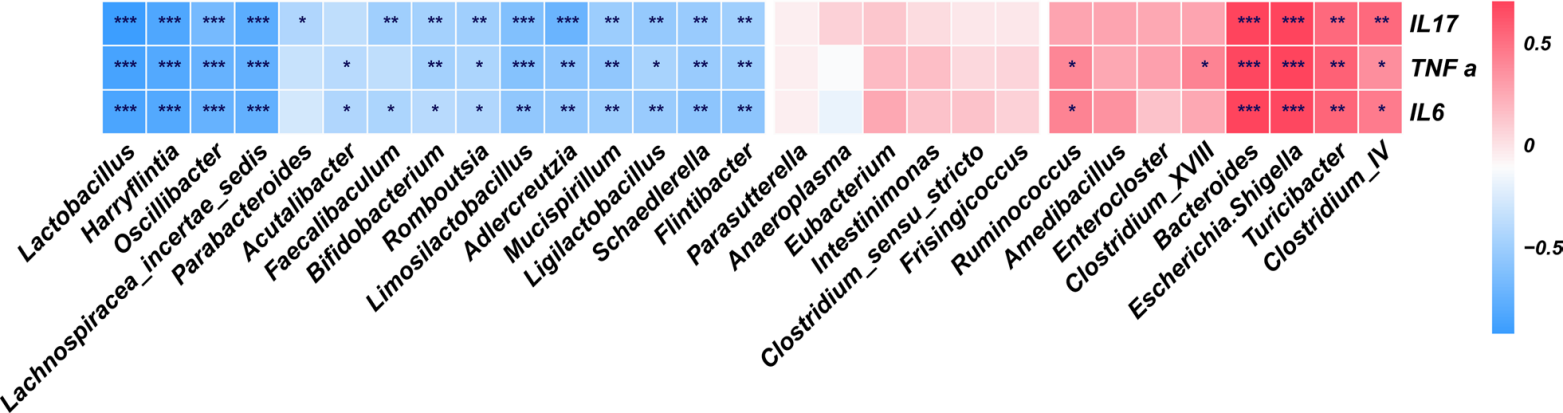
Correlation analysis between gut microbiota and inflammatory cytokines

The gut microbiota has been shown to regulate systemic chronic inflammation in a variety of diseases. Therefore, potential correlations between the fecal microbiota and the inflammatory cytokines TNF-α, IL-6, and IL-17 were analyzed. Analysis of correlations between the gut microbiota and serum cytokine concentrations revealed inverse correlations between the relative abundances of *Romboutsia* and *Adlercreutzia* and the concentrations of TNF-α, IL-6, and IL-17 (Fig. 7). Negative correlations were also identified between these cytokines and beneficial bacterial genera such as *Lactobacillus*, *Faecalibaculum*, *Bifidobacterium*, and *Ligilactobacillum*, while positive correlations were identified between the cytokines and the relative abundances of opportunistic pathogens such as *Escherichia*/*Shigella* species.

## Discussion

Currently, most probiotics are sold as food or nutritional supplements. Little research has been conducted to demonstrate the use of these probiotics to prevent or treat specific diseases. BLa80 is a commonly used probiotic in China. In this study, we investigated the potentially ameliorative effect of *B. lactis* BLa80 on DSS-induced acute UC in mice and found that *B. lactis* BLa80 promoted the healing of DSS-induced UC in mice, as shown by the decrease in DAI, mitigation of colon length shortening, and reversal of body weight loss. Histological analysis also confirmed that *B. lactis* BLa80 intervention alleviated intestinal inflammation in the UC model mice. We further observed a positive modulatory effect of *B.* lactis BLa80 on the gut microbiota in mice with UC. Our results are consistent with recent findings showing that *B. lactis* XLTG11 can attenuate DSS-induced UC in a process associated with inhibition of signalling through the TLR4/MYD88/NF-κB pathway, modulation concentrations of inflammatory cytokines, and remodelling of the gut microbiota (Wang et al. 2021).

Various cytokines are involved in regulating intestinal mucosal inflammation and intestinal epithelial integrity (Sun et al. 2015; Yu et al. 2012). The balance of proinflammatory and anti-inflammatory cytokines in the colonic mucosa forms the basis for a stable intestinal environment, and disturbance of this balance facilitates the overproduction of proinflammatory cytokines, triggering a disease state in people with IBD (Casini-Raggi et al. 1995; Xavier and Podolsky 2007). The interaction between the gut microbiota and the host immune system is an important factor in balancing and resolving inflammation. Some studies have suggested that modulation of the gut microbiota can be used as a therapeutic strategy to manage several gastrointestinal disorders, including IBD (Dotan and Rachmilewitz 2005; Vieira et al. 2016). We found that when treated with BLa80, UC model mice exhibited decreased serum concentrations of the proinflammatory cytokines TNF-α, IL-6, and IL-17. Therefore, we speculate that the anti-colitis effect of *B. lactis* BLa80 may be attributable to the regulation of inflammatory mediators. An intact intestinal barrier is essential for maintaining a healthy intestine and preventing pathogen colonisation. DSS-induced acute UC can increase intestinal permeability, damage intestinal epithelial cells, and disrupt the function of tight junction proteins in the intestinal barrier. Increased intestinal permeability leads to increased blood concentrations of lipopolysaccharide, which triggers an inflammatory response (Lakhan and Kirchgessner 2010; Lambert et al. 2002). The decreased serum concentrations of inflammatory cytokines observed after *B. lactis* BLa80 treatment provide explanations for the anti-colitis effect of *B. lactis* BLa80.

The intestinal microbiota plays an important role in maintaining physiological functions of the host. Whether intestinal microbial dysbiosis is a cause or a consequence of intestinal inflammation in mice with colitis remains controversial (Dotan and Rachmilewitz 2005; Vieira et al. 2016). Using high-throughput pyrosequencing analysis, we confirmed the regulatory effect of *B. lactis* BLa80 on the gut microbiota in UC mice. Specifically, *B. lactis* BLa80 appeared to change the composition of gut microbiota by selectively increasing the abundances of beneficial bacteria such as *Romboutsia* and *Adlercreutzia*, suggesting that BLa80 provides considerable positive support for intestinal homeostasis. Recent studies have shown that species of the genus *Romboutsia* have multiple metabolic capacities such as carbohydrate utilization, individual amino acids fermentation, anaerobic respiration, and end products of microbial metabolism (Gerritsen 2015; Gerritsen et al. 2019). Recent studies have also shown the apparent depletion of *Romboutsia* in cancerous mucosa and adenomatous polyps (Mangifesta et al. 2018). *Adlercreutzia*, a genus in the phylum *Actinobacteria*, comprises exclusively anaerobic equol-producing bacteria (Takahashi et al. 2021). Studies have shown that in the human intestine, *Adlercreutzia* species can convert resveratrol to dihydroresveratrol (Bode et al. 2013). Influenza A virus-infected mice treated with *Lactobacillus mucosae* 1025 and *Bifidobacterium pumilus* CCFM1026, exhibited increases in the relative abundance of *Adlercreutzia*, *Lactobacillus*, and *Bifidobacterium*, and modulation of short-chain fatty acid metabolism, leading to enhanced production of butyrate (Lu et al. 2021). We thus consider that the anti-inflammatory effect of BLa80 may be associated with changes in the gut microbiota.

We did not detect a significant increase in the abundance of *Bifidobacterium* in the gut microbiota in the BLa80 group. Ekmekciu et al*.* observed that the abundance of *Bifidobacterium* remained unchanged after treatment with VSL3 (Ekmekciu et al. 2017). Celiberto et al. did not observe significant changes in the abundance of *Bifidobacterium* in rats treated with *Bifidobacterium longum* ATCC15707 and *Enterococcus faecium* CRL183 (Celiberto et al. 2017). Our findings are consistent with these reports. We observed that DSS administration led to the disturbance of the gut microbiota in mice, as indicated by the significant enrichment of *Escherichia*/*Shigella* in the UC group compared with the CTL group, whereas BLa80 reshaped the intestinal microbiota and promoted the growth of beneficial bacteria, including the genera *Romboutsia* and *Adlercreutzia*. Most IBD studies have shown an increased abundance of *Enterobacteriaceae* species (Gevers et al. 2014; Mirsepasi-Lauridsen et al. 2019; Petito et al. 2019). In this study, *B. lactis* BLa80 effectively inhibited the colonisation of opportunistic pathogens and increased the population of beneficial bacteria in the mouse colon.

At the microbial function level, we observed significant increases in the metabolic pathways related to carbohydrate metabolism and glycan biosynthesis and in the metabolism of intestinal bacteria in the UC group. We speculate that these increases may be related to increases in the abundance of microbes capable of degrading glycans in the mucus secreted by the host, consistent with the disruption of the intestinal mucosal barrier in UC mice. Therefore, microorganisms that can utilize these endogenous glycans efficiently may have different degrees of impact on colon health, especially in states of host dysfunction (Koropatkin et al. 2012). The ability of BLa80 intervention to significantly reverse these changes associated with UC suggests that BLa80 helps to regulate the gut microbiota and improve the intestinal mucosal barrier.

This study focused on a single experiment to assess the therapeutic effect of BLa80 on acute colitis, and therefore, we cannot provide sufficient information about the reproducibility of our findings. Additionally, the potential link between the severity of acute colitis and the gut microbiota has not been analyzed. Finally, the mechanism by which *B. lactis* BLa80 inhibits acute inflammation in DSS-induced UC models has not been fully elucidated and may involve targeting molecules or related signal pathways.

In summary, the intervention with *B. lactis* BLa80 can significantly alleviate symptoms of DSS-induced acute UC in mice. *B. lactis* BLa80 not only alleviated macroscopic pathological findings, as indicated by the improved DAI, but also alleviated DSS-induced inflammation. The results of this study suggest that *B. lactis* BLa80 intervention enhances the stability of the gut microbiota by selectively promoting the growth of beneficial bacteria, including the genera *Romboutsia* and *Adlercreutzia*.

## Data Availability

Data will be made available on reasonable request.

## References

[CR1] Abreu MT (2002). The pathogenesis of inflammatory bowel disease: translational implications for clinicians. Curr Gastroenterol Rep.

[CR2] Aggeletopoulou I, Konstantakis C, Assimakopoulos SF, Triantos C (2019). The role of the gut microbiota in the treatment of inflammatory bowel diseases. Microb Pathog.

[CR3] Bode LM, Bunzel D, Huch M, Cho GS, Ruhland D, Bunzel M, Bub A, Franz CM, Kulling SE (2013). In vivo and in vitro metabolism of trans-resveratrol by human gut microbiota. Am J Clin Nutr.

[CR4] Casini-Raggi V, Kam L, Chong Y, Fiocchi C, Pizarro TT, Cominelli F (1995). Mucosal imbalance of IL-1 and IL-1 receptor antagonist in inflammatory bowel disease. A novel mechanism of chronic intestinal inflammation. J Immunol.

[CR5] Celiberto LS, Bedani R, Dejani NN, Ivo de Medeiros A, Sampaio Zuanon JA, Spolidorio LC, Tallarico Adorno MA, Amâncio Varesche MB, Carrilho Galvão F, Valentini SR, Font de Valdez G, Rossi EA, Cavallini DCU (2017). Effect of a probiotic beverage consumption (*Enterococcus faecium* CRL 183 and *Bifidobacterium longum* ATCC 15707) in rats with chemically induced colitis. PLoS ONE.

[CR6] Chae JM, Heo W, Cho HT, Lee DH, Kim JH, Rhee MS, Park TS, Kim YK, Lee JH, Kim YJ (2018). effects of orally-administered *Bifidobacterium*
*animalis* subsp. *lactis* strain BB12 on dextran sodium sulfate-induced colitis in mice. J Microbiol Biotechnol.

[CR7] Cheifetz AS (2013). Management of active Crohn disease. JAMA.

[CR8] Debnath T, Kim DH, Lim BO (2013). Natural products as a source of anti-inflammatory agents associated with inflammatory bowel disease. Molecules.

[CR9] Dotan I, Rachmilewitz D (2005). Probiotics in inflammatory bowel disease: possible mechanisms of action. Curr Opin Gastroenterol.

[CR10] Edgar RC (2016). SINTAX: a simple non-Bayesian taxonomy classifier for 16S and ITS sequences. Biorxiv.

[CR11] Ekmekciu I, von Klitzing E, Fiebiger U, Neumann C, Bacher P, Scheffold A, Bereswill S, Heimesaat MM (2017). The probiotic compound VSL#3 modulates mucosal, peripheral, and systemic immunity following murine broad-spectrum antibiotic treatment. Front Cell Infect Microbiol.

[CR12] Gerritsen J (2015). The genus *Romboutsia*: genomic and functional characterization of novel bacteria dedicated to life in the intestinal tract.

[CR13] Gerritsen J, Hornung B, Ritari J, Paulin L, Rijkers GT, Schaap PJ, de Vos WM, Smidt H (2019). A comparative and functional genomics analysis of the genus *Romboutsia* provides insight into adaptation to an intestinal lifestyle. BioRxiv.

[CR14] Gevers D, Kugathasan S, Denson LA, Vázquez-Baeza Y, Van Treuren W, Ren B, Schwager E, Knights D, Song SJ, Yassour M, Morgan XC, Kostic AD, Luo C, González A, McDonald D, Haberman Y, Walters T, Baker S, Rosh J, Stephens M, Heyman M, Markowitz J, Baldassano R, Griffiths A, Sylvester F, Mack D, Kim S, Crandall W, Hyams J, Huttenhower C, Knight R, Xavier RJ (2014). The treatment-naive microbiome in new-onset Crohn’s disease. Cell Host Microbe.

[CR15] Gonçalves P, Araújo JR, Di Santo JP (2018). A cross-talk between microbiota-derived short-chain fatty acids and the host mucosal immune system regulates intestinal homeostasis and inflammatory bowel disease. Inflamm Bowel Dis.

[CR16] Hassan SM, Hassan AH (2018). The possibility of using shogaol for treatment of ulcerative colitis. Iran J Basic Med Sci.

[CR17] Hill C, Guarner F, Reid G, Gibson GR, Merenstein DJ, Pot B, Morelli L, Canani RB, Flint HJ, Salminen S (2014). The International Scientific Association for Probiotics and Prebiotics consensus statement on the scope and appropriate use of the term probiotic. Nat Rev Gastroenterol Hepatol.

[CR18] Howarth GS (2008). Inflammatory bowel disease, a dysregulated host-microbiota interaction: are probiotics a new therapeutic option?.

[CR19] Khan I, Ullah N, Zha L, Bai Y, Khan A, Zhao T, Che T, Zhang C (2019). Alteration of gut microbiota in inflammatory bowel disease (IBD): cause or consequence? IBD treatment targeting the gut microbiome. Pathogens.

[CR20] Khor B, Gardet A, Xavier RJ (2011). Genetics and pathogenesis of inflammatory bowel disease. Nature.

[CR21] Koropatkin NM, Cameron EA, Martens EC (2012). How glycan metabolism shapes the human gut microbiota. Nat Rev Microbiol.

[CR22] Kumar CS, Reddy KK, Boobalan G, Reddy AG, Chowdhary CSR, Vinoth A, Jayakanth K, Rao GS (2017). Immunomodulatory effects of *Bifidobacterium bifidum* 231 on trinitrobenzenesulfonic acid-induced ulcerative colitis in rats. Res Vet Sci.

[CR23] Lakatos PL, Lakatos L (2008). Ulcerative proctitis: a review of pharmacotherapy and management. Expert Opin Pharmacother.

[CR24] Lakhan SE, Kirchgessner A (2010). Neuroinflammation in inflammatory bowel disease. J Neuroinflammation.

[CR25] Lambert G, Gisolfi CV, Berg DJ, Moseley PL, Oberley LW, Kregel KC (2002). Selected contribution: hyperthermia-induced intestinal permeability and the role of oxidative and nitrosative stress. J Appl Physiol.

[CR26] Langille MGI, Zaneveld J, Caporaso JG, McDonald D, Knights D, Reyes JA, Clemente JC, Burkepile DE, Vega Thurber RL, Knight R, Beiko RG, Huttenhower C (2013). Predictive functional profiling of microbial communities using 16S rRNA marker gene sequences. Nat Biotechnol.

[CR27] Logan AC, Katzman M (2005). Major depressive disorder: probiotics may be an adjuvant therapy. Med Hypotheses.

[CR28] Lu W, Fang Z, Liu X, Li L, Zhang P, Zhao J, Zhang H, Chen W (2021). The potential role of probiotics in protection against influenza a virus infection in mice. Foods.

[CR29] Mangifesta M, Mancabelli L, Milani C, Gaiani F, de’Angelis N, de’Angelis GL, van Sinderen D, Ventura M, Turroni F (2018). Mucosal microbiota of intestinal polyps reveals putative biomarkers of colorectal cancer. Sci Rep.

[CR30] Marchesi JR, Holmes E, Khan F, Kochhar S, Scanlan P, Shanahan F, Wilson ID, Wang Y (2007). Rapid and noninvasive metabonomic characterization of inflammatory bowel disease. J Proteome Res.

[CR31] Mirsepasi-Lauridsen HC, Vallance BA, Krogfelt KA, Petersen AM (2019). *Escherichia coli* pathobionts associated with inflammatory bowel disease. Clin Microbiol Rev.

[CR32] Neu J (2019). Multiomics-based strategies for taming intestinal inflammation in the neonate. Curr Opin Clin Nutr Metab Care.

[CR33] Norouzinia M, Chaleshi V, Alizadeh AHM, Zali MR (2017). Biomarkers in inflammatory bowel diseases: insight into diagnosis, prognosis and treatment. Gastroenterol Hepatol Bed Bench.

[CR34] Oksanen J, Blanchet F, Friendly M, Kindt R, Legendre P, McGlinn D, Minchin P, O’Hara R, Simpson G, Solymos P (2019). vegan: community ecology package.

[CR35] Parks DH, Tyson GW, Hugenholtz P, Beiko RG (2014). STAMP: statistical analysis of taxonomic and functional profiles. Bioinformatics.

[CR36] Petito V, Fiore L, Lopetuso LR, Scaldaferri F (2019). Commentary to “Safety, Clinical response, and microbiome findings following fecal microbiota transplant in children with inflammatory bowel disease”. Inflamm Bowel Dis.

[CR37] Segata N, Izard J, Waldron L, Gevers D, Miropolsky L, Garrett WS, Huttenhower C (2011). Metagenomic biomarker discovery and explanation. Genome Biol.

[CR38] Sergent T, Piront N, Meurice J, Toussaint O, Schneider YJ (2010). Anti-inflammatory effects of dietary phenolic compounds in an in vitro model of inflamed human intestinal epithelium. Chem Biol Interact.

[CR39] Shadnoush M, Hosseini RS, Mehrabi Y, Delpisheh A, Alipoor E, Faghfoori Z, Mohammadpour N, Moghadam JZ (2013). Probiotic yogurt affects pro-and anti-inflammatory factors in patients with inflammatory bowel disease. Iran J Pharm Res.

[CR40] Singh AK, Hertzberger RY, Knaus UG (2018). Hydrogen peroxide production by *Lactobacilli* promotes epithelial restitution during colitis. Redox Biol.

[CR41] Sun M, He C, Cong Y, Liu Z (2015). Regulatory immune cells in regulation of intestinal inflammatory response to microbiota. Mucosal Immunol.

[CR42] Takahashi H, Yang J, Yamamoto H, Fukuda S, Arakawa K (2021). Complete genome sequence of *Adlercreutzia*
*equolifaciens* subsp. *celatus* DSM 18785. Microbiol Resour Announc.

[CR43] Team RC (2013). R: a language and environment for statistical computing.

[CR44] Veerappan GR, Betteridge J, Young PE (2012). Probiotics for the treatment of inflammatory bowel disease. Curr Gastroenterol Rep.

[CR45] Vieira AT, Fukumori C, Ferreira CM (2016). New insights into therapeutic strategies for gut microbiota modulation in inflammatory diseases. Clin Transl Immunol.

[CR46] Wang K, Jin X, You M, Tian W, Leu RKL, Topping DL, Conlon MA, Wu L, Hu F (2017). Dietary propolis ameliorates dextran sulfate sodium-induced colitis and modulates the gut microbiota in rats fed a western diet. Nutrients.

[CR47] Wang N, Wang S, Xu B, Liu F, Huo G, Li B (2021). Alleviation Effects of *Bifidobacterium*
*animalis* subsp. *lactis* XLTG11 on dextran sulfate sodium-induced colitis in mice. Microorganisms.

[CR48] Wickham H (2017). ggplot2-elegant graphics for data analysis (2nd edition). J Stat Softw.

[CR49] Xavier RJ, Podolsky D (2007). Unravelling the pathogenesis of inflammatory bowel disease. Nature.

[CR50] Xie Y, Guo Q, Li S, Liu M, Zhang Q, Xu Z, Sun H (2017). Anti-inflammatory properties of *Bifidobacterium longum* expressing human manganese superoxide dismutase using the TNBS-induced rats model of colitis. J Microbiol Biotechnol.

[CR51] Yu LCH, Wang JT, Wei SC, Ni YH (2012). Host-microbial interactions and regulation of intestinal epithelial barrier function: from physiology to pathology. World J Gastrointest Pathophysiol.

[CR52] Zhao TS, Xie LW, Cai S, Xu JY, Zhou H, Tang LF, Yang C, Fang S, Li M, Tian Y (2021). Dysbiosis of gut microbiota is associated with the progression of radiation-induced intestinal injury and is alleviated by oral compound probiotics in mouse model. Front Cell Infect Microbiol.

